# Modular Control of Treadmill *vs* Overground Running

**DOI:** 10.1371/journal.pone.0153307

**Published:** 2016-04-11

**Authors:** Anderson Souza Oliveira, Leonardo Gizzi, Shahin Ketabi, Dario Farina, Uwe Gustav Kersting

**Affiliations:** 1 Department of Mechanical and Manufacturing Engineering, Aalborg University, Aalborg, Denmark; 2 Institute of Neurorehabilitation Systems, Bernstein Focus Neurotechnology Göttingen, Bernstein Center for Computational Neuroscience, University Medical Center Göttingen, Georg-August University, Göttingen, Germany; 3 Department of Health Science and Technology, Aalborg University, Aalborg, Denmark; Shanghai Jiao Tong University, CHINA

## Abstract

Motorized treadmills have been widely used in locomotion studies, although a debate remains concerning the extrapolation of results obtained from treadmill experiments to overground locomotion. Slight differences between treadmill (TRD) and overground running (OVG) kinematics and muscle activity have previously been reported. However, little is known about differences in the modular control of muscle activation in these two conditions. Therefore, we aimed at investigating differences between motor modules extracted from TRD and OVG by factorization of multi-muscle electromyographic (EMG) signals. Twelve healthy men ran on a treadmill and overground at their preferred speed while we recorded tibial acceleration and surface EMG from 11 ipsilateral lower limb muscles. We extracted motor modules representing relative weightings of synergistic muscle activations by non-negative matrix factorization from 20 consecutive gait cycles. Four motor modules were sufficient to accurately reconstruct the EMG signals in both TRD and OVG (average reconstruction quality = 92±3%). Furthermore, a good reconstruction quality (80±7%) was obtained also when muscle weightings of one condition (either OVG or TRD) were used to reconstruct the EMG data from the other condition. The peak amplitudes of activation signals showed a similar timing (pattern) across conditions. The magnitude of peak activation for the module related to initial contact was significantly greater for OVG, whereas peak activation for modules related to leg swing and preparation to landing were greater for TRD. We conclude that TRD and OVG share similar muscle weightings throughout motion. In addition, modular control for TRD and OVG is achieved with minimal temporal adjustments, which were dependent on the phase of the running cycle.

## Introduction

Motorized treadmills have been widely used in locomotion studies ranging from basic physiology to motor rehabilitation. Treadmills offer the possibility of implementing standardized and reproducible test conditions. However, a long debate has been raised concerning the transfer of results obtained from treadmill experiments to overground locomotion. Discussions were initiated by Nelson et al. [1], who supported the opinion of similar physical demands for treadmill and overground running. This suggestion was partly contradicted by experiments showing slight differences in spatiotemporal parameters of running, such as stride length and stride rate [1–4] and reduced peak ground reaction forces during treadmill running [2,5]. Concerning kinematics, the literature shows inconsistent evidence on whether treadmill and overground running are identical. Some studies have shown similar running patterns between conditions [2,3], whereas other studies revealed significant differences in a few variables, such as lumbar and pelvic positioning at initial contact [3,6], ankle excursion and ankle eversion [6] and peak knee sagittal plane angle [2]. Based on previous studies it may not be possible to state that treadmill and overground running are kinematically identical, and it remains to be shown if motor control strategies are similar between the two environmental conditions.

Surface electromyography (EMG) provides an indirect estimation of neural input to the muscles [7], but only a few studies have compared muscle activation during treadmill and overground running. Wank et al. [4] have reported similar results between these two running conditions. Conversely, Baur and Hirschmüller [8] have shown specific changes, such as an earlier and longer EMG activity for peroneus longus, and a reduced amplitude for soleus during treadmill running. These previous studies analysed surface EMG from lower limb muscles separately, and the conclusions were restricted to the independent results of individual muscles. Studies on selective activation have demonstrated that the neural pathways in primates do not allow for an activation of single muscles or motor units, and that the central nervous system learns to control specific degrees of freedom with training [9,10]. Although analysing EMG for each muscle separately is common practice in biomechanical analyses, it potentially neglects the interactions resulting from the underlying control structures. Therefore a multi muscular approach is envisaged.

Human locomotion is considered a natural motor behaviour, and as such the central nervous system acts by controlling groups of muscles related to specific mechanical requirements [7,11]. The central nervous system controls locomotion, as well as other complex motor behaviour, by means of a low-dimensional set of muscle synergies or motor modules—defined as a selection of muscles that are recruited in a specific timing sequence with fixed relative activation [7,12,13]. These motor modules act at the spinal cord triggering selected central pattern generators that evoke specific motor behaviour [7,14–16]. Recent investigations have broadly applied this methodology to explain basic modulation of running [12], inter-individual variability during cycling [17] and gymnastics [18], expertise level in rowing [19] and (external) work production during side-step cutting manoeuvres [20]. Similar to walking, the patterns of muscle activation for running have been described by burst-like activation of motor modules encoded at the spinal level [12,13]. The extraction of motor modules from surface EMG is suitable for investigating potential differences in motor control strategies if running is performed under different conditions, such as treadmill and overground locomotion.

The temporal properties of motor modules related to locomotion may be influenced by biomechanical task constraints [21,22]. Oliveira et al. [13] demonstrated that power generation for whole-body deceleration and acceleration is correlated to the timing of motor modules during stance. Furthermore, Martino et al. [21] demonstrated that walking in unstable conditions (i.e., slippery ground, narrow beam) induced substantial differences in the timing properties of motor modules, which were related to sensorial inputs during the execution of the motor program. In fact, walking on unstable surfaces increases the activation within the sensorimotor cortex [23]. These results suggest that the central nervous system may tune the recruitment of existing motor modules to accommodate variable biomechanical demands guided by sensorial and/or sensorimotor processing [20,21,23,24]. Moreover, adjustments in muscle recruitment to perform treadmill running may be based on sensorial information towards optimizing postural stability within the confined area of the treadmill. Additional tuning of muscle activation might be necessary for adjusting stride length and duration, as well as the optimal body position for performing comfortable running on the constantly moving belt.

In this study, we aimed at investigating whether motor modules and their timing properties are different between treadmill and overground running. We first hypothesized that treadmill and overground running present similar modular organization, as the neural control of locomotion in humans is robust and may be strongly encoded at the spinal level [12,25]. Second, treadmill running may require specific postural adjustments due to the constantly moving belt and confined space individuals are exposed to. Thus, we hypothesized that temporal properties of muscle activation during stance and swing phases are differently adjusted for treadmill and overground running regarding altered sensorial inputs and stride variability.

## Experimental Procedures

### Subjects

Twelve healthy men (age: 28±4 yrs; body mass: 80.8±8 kg; stature: 178±4 cm) volunteered for the experiment. All subjects were practicing running two to three times/week, and had previous experience in treadmill running. All subjects were rearfoot runners, therefore the maximum acceleration for each gait cycle was linked to initial heel contact to the floor. One subject was left-dominant whilst all others were right-dominant. Exclusion criteria included any history of knee or ankle ligament injury, current lower-extremity injury, recent (within 6 months) low back injury, or vestibular dysfunction. All subjects provided written informed consent before participation and the procedures were approved by the ethical committee of Northern Jutland (N-20130015).

### Experimental Setup

In a single session, subjects initially familiarized themselves to the treadmill (WoodwayPro, Foster Court Waukesha, USA) by walking and running for 5 minutes. Subsequently, we determined their preferred running speed using an adapted protocol based on Jason et al. [26]. Briefly, the test started at a low running speed (~1.5 m/s) which was increased by ~0.1 m/s every 20 s with the speed display made invisible for the participants. We increased the speed until the subjects indicated running at their preferred speed. After one minute rest, running started at a speed ~0.6 m/s faster than the previously reported preferred speed. From there, we gradually decreased the speed by ~0.1 m/s every 20 s until the subject indicated again haven hit the preferred speed. We repeated this procedure until we found a close match (less than 0.2 m/s difference between the two reported speed) between both approach directions. We defined the preferred speed as the average of the closest preferred speeds. We found similar preferred speed in the first and second attempt for all subjects with the total duration of this protocol being less than ~10 minutes.

#### Running protocol

After a 2-minute rest period from determining the preferred speed, the subjects ran on the treadmill at this speed for 6 minutes during which we acquired stride frequency (by means of counting the number of steps) and surface EMG from the last 2 minutes. In this way, the recorded data were representing a period in which participants were used to the speed and the treadmill, minimizing potential influences of adaptation [27,28]. In a second task, the subjects were running overground along a 75-m straight indoor corridor. They were asked to run along the corridor while keeping the preferred speed, being guided by a metronome set at the same stride frequency as on the treadmill. Pilot trials without using the metronome showed that the stride frequency from overground and treadmill running were similar at comparable speeds (difference <0.2 m/s). Moreover, the use of the target stride frequency from treadmill running helped subjects to maintain their speed during the overground running protocol. After 3–5 minutes of familiarization to the environment, stride frequency and speed, we recorded two minutes of continuous surface EMG by running along the corridor five to six times at an average speed within ±0.2 m/s of the preferred speed. We determined running speed during the overground condition by the time spent to cover the central 60 m of the 75 m corridor with the acceleration and deceleration phases being excluded. Equivalently, we excluded from the analysis the recordings in which subjects lost pace and/or showed no constant running pattern. The order of the tasks was not randomized, with treadmill data being collected first for all subjects. Running has been demonstrated as being a strongly automated motor behavior [12] while we only included active runners with previous experience in treadmill running on this study. Therefore, we did not expect any learning or adaptation effects when switching between tasks. Moreover, subjects ran no longer than 6–10 minutes in each condition with resting periods of 1–2 minutes after every recording being administered throughout the whole experiment. Basset et al. [29] have shown similar oxygen consumption for treadmill and overground running at similar speeds. Therefore, it could be assumed that fatigue effects would not have an influence on the results of this study.

### Data collection

**Fig 1:**
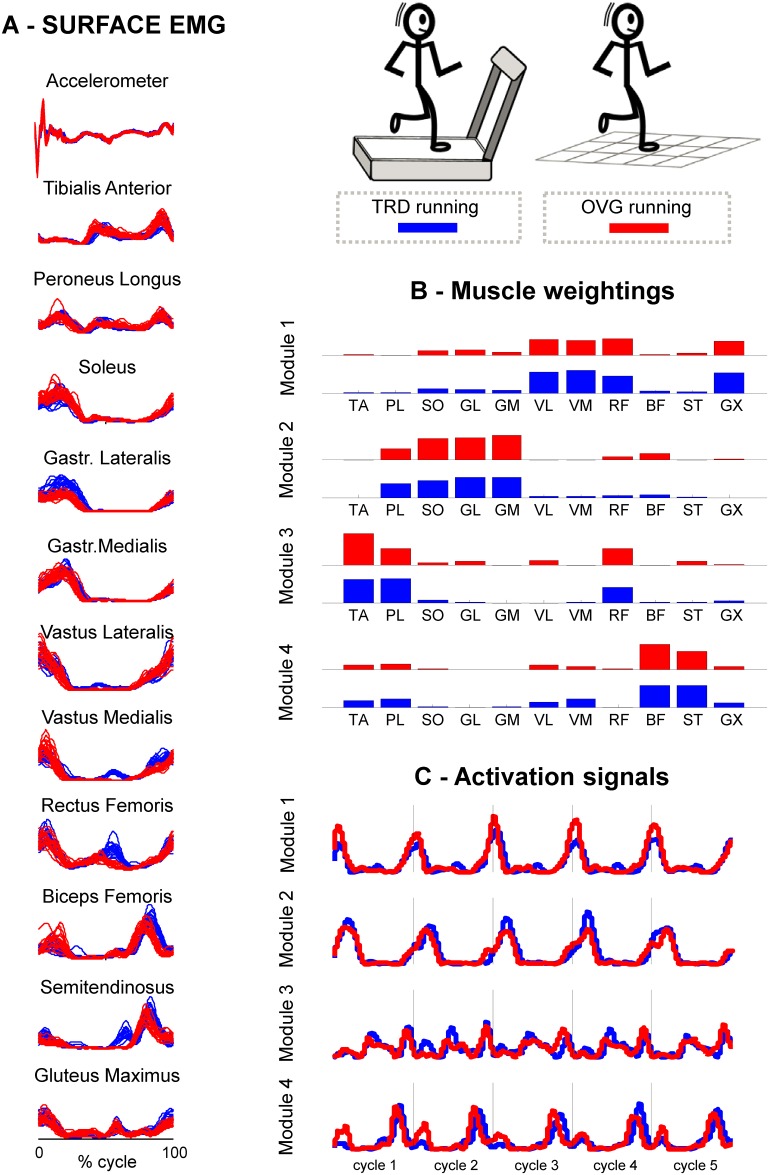
Experimental design. Illustration of experimental design involving treadmill (*blue*) and overground running (*red*) from a representative subject. Tibia vertical acceleration used to identify gait cycles, and surface EMG are plotted in (A) (raw data for acceleration, envelope for sEMG). The gait cycles were defined from one left foot strike to the following left foot strike. The motor modules extracted from the concatenated surface EMG are represented as “muscle weightings” (panel B) and “activation signals “(panel C), in this case 5 consecutive gait cycles are reported. It is worth noting that by concatenating several gait cycles we accounted the inter-cycle variability in the analysis.

For recording EMG signals, we used bipolar derivations with pairs of Ag/AgCl electrodes (AmbuNeuroline 720 01-K/12; Ambu, Ballerup, Denmark) with 22 mm of center-to-center spacing. Prior to electrode placement, we shaved and lightly abraded the skin. We recorded the EMG signals from the following muscles ipsilaterally (left side) according to Barbero et al. [30]: tibialis anterior (TA), peroneus longus (PL), soleus (SO), gastrocnemius lateralis (GL), gastrocnemius medialis (GM), vastus lateralis (VL), vastus medialis (VM), rectus femoris (RF), biceps femoris (BF), semitendinosus (ST), and gluteus maximus (GX). We placed a reference electrode on the left tibia, subsequently attaching a uniaxial accelerometer (Biovision ACC, Wehrheim, Germany) to the left tibia using surgical tape (Fixomul stretch Beiersdorf, North Rhyde, NSW). We carefully fixed both sensor and cable in order to minimize artifacts during running. For this experiment, we recorded accelerometry and EMG simultaneously using a portable EMG system (Biovision, Wehrheim, Germany) stored in a backpack together with a mini-computer, for both treadmill and overground conditions. The EMG signals were sampled at 2,000 Hz (12 bits per sample), band-pass filtered (second-order, zero lag Butterworth, bandwidth 10–500 Hz) and recorded on the computer’s storage medium for off-line analysis. Fig 1A shows example EMG traces from a representative subject.

### Data analysis

#### Accelerometry

We low-pass filtered the accelerometer data (60Hz) and determined gait cycles from an adaptation of the method described in Selles et al., [31]. Briefly, we identified minimum acceleration instances throughout the continuous time-series. Running induces substantial changes in acceleration at foot strike, allowing for a first estimation of the stride duration using the minimum acceleration [32]. We subsequently differentiated the filtered accelerometer data and each foot strike was defined as the positive peak of the derivative immediately prior to the minimum acceleration defined previously. We excluded gait cycles showing erroneous EMG envelopes in comparison to expected curves by visual inspection. Individual gait cycles were time-normalized to 200 data points for one gait cycle. Peak negative acceleration at foot strike was determined. In addition, we calculated the inter-trial variability of acceleration patterns in two distinct phases of the running cycle: 1) the acceleration during stance, calculated from 0–35% of the running cycle [2] and 2) the acceleration during pre-landing, calculated from 91–100% of the running cycle. We defined the variability as the ratio of the standard deviation to the mean (coefficient of variation).

#### Surface EMG segmentation

We defined the segmentation for EMG factorization from the accelerometer data, from which running cycles were determined. After segmentation, the surface EMG signals from the 11 muscles were full-wave rectified, low-pass filtered (10 Hz) and time-normalized in order to obtain 200 data points for one gait cycle [33,34].

#### EMG analysis—peak and integrated EMG

The first part of our analysis involved the comparison of the amplitude of the EMG signals in the two conditions using a single-muscle analysis. For treadmill running, we selected 20 consecutive gait cycles from the last 60 seconds of EMG recordings. In the case of overground running, we selected 20 consecutive gait cycles from the attempt in which subjects covered the corridor distance at a speed closest to the target speed. For each muscle of each subject, we used the averaged EMG amplitude from all 20 gait cycles from the treadmill condition to normalize EMG curves from both treadmill and overground running. Subsequently, we calculated normalized peak EMG, and integrated EMG from the entire cycles for each muscle separately for the two conditions.

#### EMG analysis—motor modules

The second part of the analysis involved the factorization of the EMG matrices into motor modules. We performed the analysis of motor modules using the original segmented, filtered and time-normalized EMG cycles. For each subject and condition, we concatenated the 20 consecutive time-normalized EMG envelopes obtained from the raw EMG through band pass filtering, rectification and low pass filtering, in order to preserve all variability contained in the task [35]. To account for differences across subjects, we normalized all the concatenated time series in amplitude from 0 to 1 (1 = overall maximum amplitude) for each recorded muscle. We then applied non-negative matrix factorization [36] to the matrix of 11 muscles x 20 running cycles of 200 samples, for each subject (matrix dimensions 11 x 20 x 200) (Fig 1B and 1C).

#### Motor modules model

The EMG signals recorded from *M* muscles were indicated as:

$$X(k)\;=\;{\left[x(k),\;x(k),\ldots ,\;x_{M}(k)\right]}^{T}$$ (1)

where *x*_*M*_*(k)* is the activity of the *m*th muscle at the time instant *k*. The activation signals *P(k)* were indicated as (*N<M*):

$$P(k)\;=\;{\left[p_{1}(k),\;p_{2}(k),\ldots ,\;p_{N})k)\right]}^{T}$$ (2)

The relation between *X(k)* and *P(k)* is described as follows:

$$X(k)\approx \;X_{r}(k)\;=\;S∙P(k)$$ (3)

where *X*_*r*_*(k)* is the muscle activity vector reconstructed by the factorization. In Eq (3), the EMGs *X(k)* are obtained by linear transformation of the activation signals *P(k)* with gain factors *s*_*mn*_. The matrix whose columns were the weights of each activation signal for each muscle is denoted as *S* in Eq (3) and will be referred to as the motor module matrix [36].

#### Dimensionality

After extracting the motor modules, we compared the estimated muscular activation pattern to the recorded signal by means of the variation accounted for (VAF) value, defined as the variation that can be explained by the model: VAF = 1 –SSE/SST, where SSE (sum of squared errors) is the unexplained variation, and SST (total sum of squares) is the pooled variation of the data. The quality of EMG reconstruction increases as a function of the number of modules extracted using non-negative matrix factorization, but after a sufficient number of modules the inclusion of additional modules does not considerably improve the reconstruction quality [37]. In order to define the minimum number of modules that can reconstruct the original EMG datasets, we displayed the VAF as a function of the number of synergies (range: 1–11). We defined the optimal dimensionality as the point at which this VAF x number of synergies changes slope [37]. In addition, the number of modules must also successfully reconstruct at least 90% of the original EMG content.

#### Similarities

Similarities between muscle weightings or activation signals were calculated by the scalar product, normalized by the product of the norms of each column [13,37], which prioritizes the comparison between the shapes of vectors rather than amplitude. Similarity can vary from 0 (no curve shape matching) to 1 (perfect curve shape matching). In previous investigations, a threshold of 0.8 has been considered sufficient to define to sets of modules “similar” [13,33]. Intra-subject similarity analyses were performed for motor modules and activation signals across conditions (treadmill *vs*. overground running). Inter-subject similarities were also computed for both treadmill and overground running separately.

#### Peak of activation signals and curve subtractions

We normalized the EMG from each muscle separately for both treadmill and overground running. In this way, potential changes in the magnitude of resultant activation signals can be related to specific inter-muscular coordination strategies for each condition. In order to investigate changes in peak activation of motor modules, we determined the peak activation signal for each motor module by averaging the 20 time-normalized activation signals and computing the peak and instant of the peak of this average curve for each subject. In addition, in order to quantitatively represent potential differences for each motor module throughout the entire gait cycle across conditions, we computed the absolute values from the subtraction of the averaged treadmill running from the averaged overground running for each motor module of each subject.

#### Reconstruction using mixed non-negative matrix factorization models

**Fig 2:**
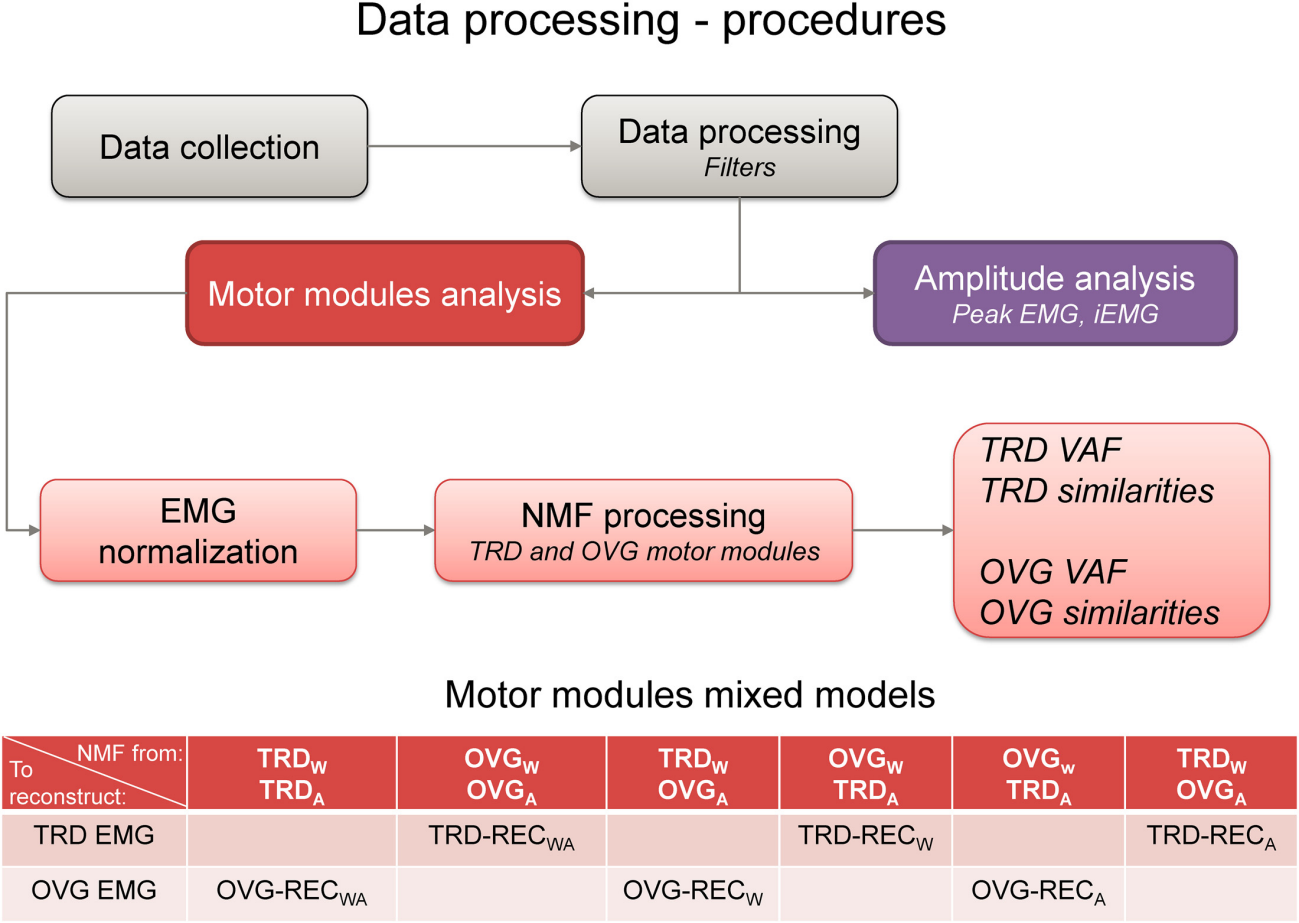
Schematic representation of EMG data processing. EMG data from treadmill (TRD) and overground running (OVG) were band-pass filtered, low-pass filtered and segmented into (20) running cycles. These segmented data were processed for the extraction of peak EMG and integrated EMG. In addition, the filtered and segmented data were processed using non-negative matrix factorization (NMF) in order to extract muscle weightings and respective activation signals for TRD and OVG separately. We reconstructed the original EMG from OVG (OVG EMG) by mixing muscle weightings and activation signals from TRD condition, generating a reconstructed OVG-REC_WA_. A second mixed model was the reconstruction of the original EMG from OVG by mixing TRD muscle weightings and OVG activation signals, generating a reconstructed OVG-REC_W_. The third mixed model was the reconstruction of the original EMG from OVG by mixing OVG muscle weightings and TRD activation signals, generating a reconstructed OVG-REC_A_. This same procedures were applied for the reconstruction of the original EMG from TRD.

In this work, we applied the concept of non-negative reconstruction [38,39] in which one of the motor module matrices (*S* or *P*) is fixed and the other matrix is updated at each iteration. In this study we replaced the matrix *S*, the matrix *P*, or both in order to compute reconstruction quality of such combinations. We assumed that if treadmill and overground running were modulated by similar motor module matrices, the use of muscle weightings and/or activation signals from overground running could be used for successfully reconstructing the EMG data from treadmill running and vice-versa. This method can assist in identifying potential sharing of muscle weighting factors and/or temporal properties between independent tasks. Therefore, in addition to the standard assessment of EMG reconstruction, we also assessed the reconstruction quality of EMG datasets in three additional combinations: 1) reconstruction of treadmill EMG using muscle weightings and activation signals from overground running (REC_WA_); 2) reconstruction of treadmill EMG using muscle weightings from overground running and activation signals from treadmill running (REC_W_) and 3) reconstruction of treadmill EMG using muscle weightings from treadmill running and activation signals from overground running (REC_A_). We applied the same methods for investigating the reconstruction quality of EMG recorded during overground running (see illustration in Fig 2 for details).

### Statistical analysis

We used Shapiro-Wilk statistic test to confirm normal distribution of the dependent variables: peak EMG, integrated EMG, acceleration during stance and pre-landing. Two-tailed paired t-tests were used on these dependent variables as well as for inter-subject similarities, for determining differences between treadmill and overground running. We calculated Cohen’s *d* effect size for all variables (0.2<*d*<0.5 = small effect, 0.5<*d*<0.8 = medium effect, d>0.8 = large effect). We tested the effects of different methods for reconstructing EMG datasets based on mixed non-negative matrix factorization models (regular reconstruction x REC_WA_ x REC_W_ x REC_A_) by using a 1-way analysis of variance, for which the VAF was the dependent variable. The significance level was set to p<0.05 for all statistical analyses. We applied Bonferroni correction to account for multiple comparisons (i.e., 11 muscles) for the analysis of peak EMG and integrated EMG. We conducted all statistical procedures using SPSS 18.0 (SPSS, Inc., Chicago, IL, USA).

## Results

Running speed and stride frequency for treadmill and overground running were not statistically different (p>0.05, Table 1). In addition, we found no significant differences for peak negative acceleration (p>0.05). However, the variability of vertical acceleration during stance and pre-landing was significantly higher during overground running (p<0.005, effect size *d =* 0.38 and 0.50 respectively).

**Table 1:** Running temporal parameters and vertical tibial acceleration. * denotes significant difference in relation to TRD running (p<0.05).

	TRD Running	OVG Running
Speed (m.s-1)	3.0±0.3	3.1±0.4
Stride Frequency (cycles/min)	82.9±4.7	83.1±5.5
Peak negative acceleration (*g*)	3.55±0.3	3.56±0.4
Variability in acceleration—stance (%)	25.1±16.8	32.2±20.5*
Variability in acceleration—pre-landing (%)	50.3±16.7	61.9±28.3*

### Electromyography

**Fig 3:**
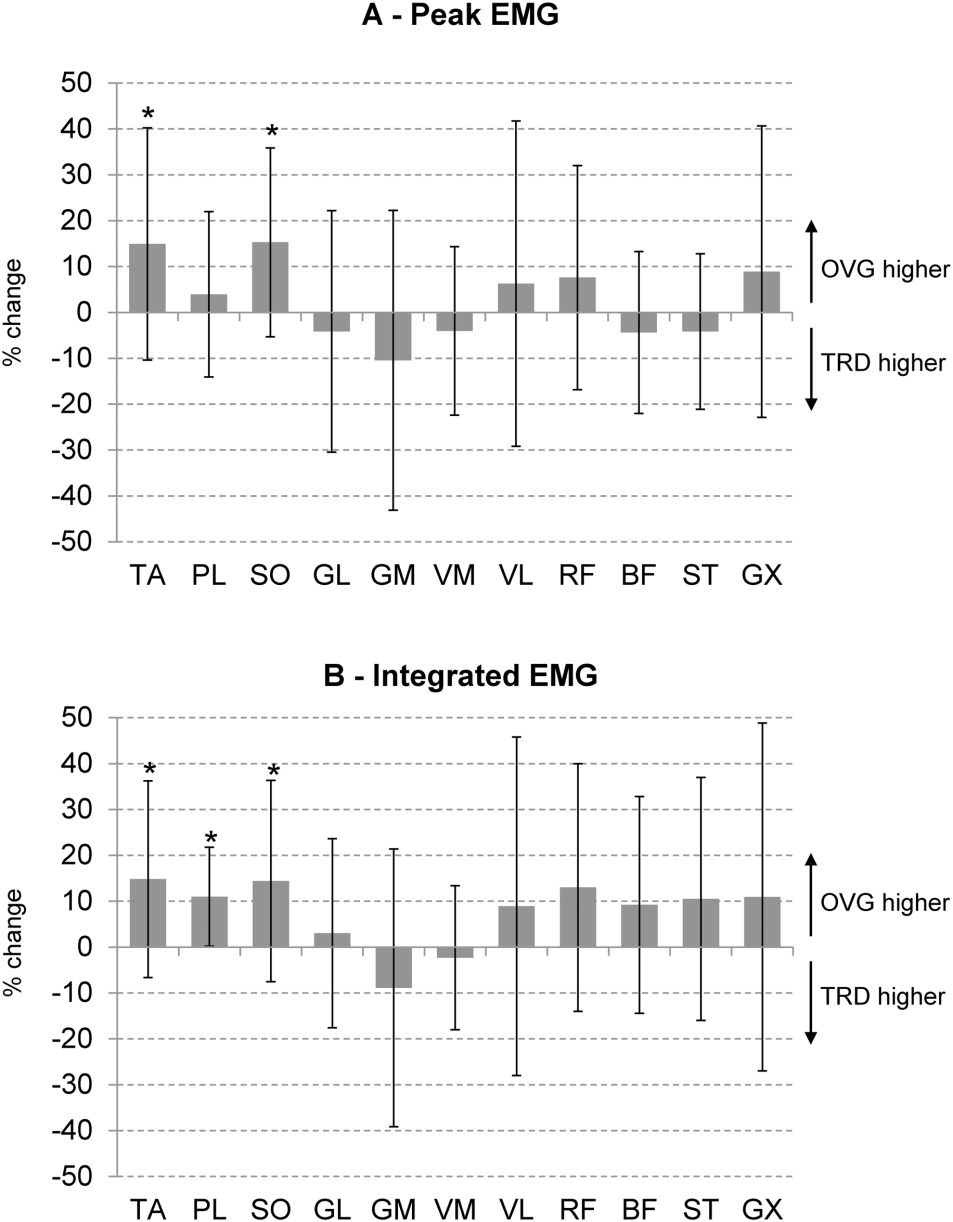
EMG Comparison of treadmill vs overground. Percentage of change between treadmill (TRD) and overground (OVG) running for the peak EMG (A) and integrated EMG (B) throughout a gait cycle. Data from OVG running were normalized by the values from TRD running. * denotes significant differences between treadmill and overground running (p<0.05).

Peak EMG (Fig 3A) and integrated EMG during the gait cycle (Fig 3B) were increased during overground running for TA and SO (>10% increase, p<0.005, effect size *d =* 0.58 and 0.82, respectively). We found a medium effect size (0.5<*d*<0.8) for integrated EMG from RF, BF and ST, however we did not find significant differences using Student t-tests for these muscles. We also found greater integrated EMG for PL, TA and SO during overground running (~10%, p<0.005, effect size *d* > 0.9). The remaining muscles showed a high inter-subject variability and small effect size (*d*<0.5). We found no further statistical differences between treadmill and overground running.

### Motor modules—dimensionality

**Fig 4:**
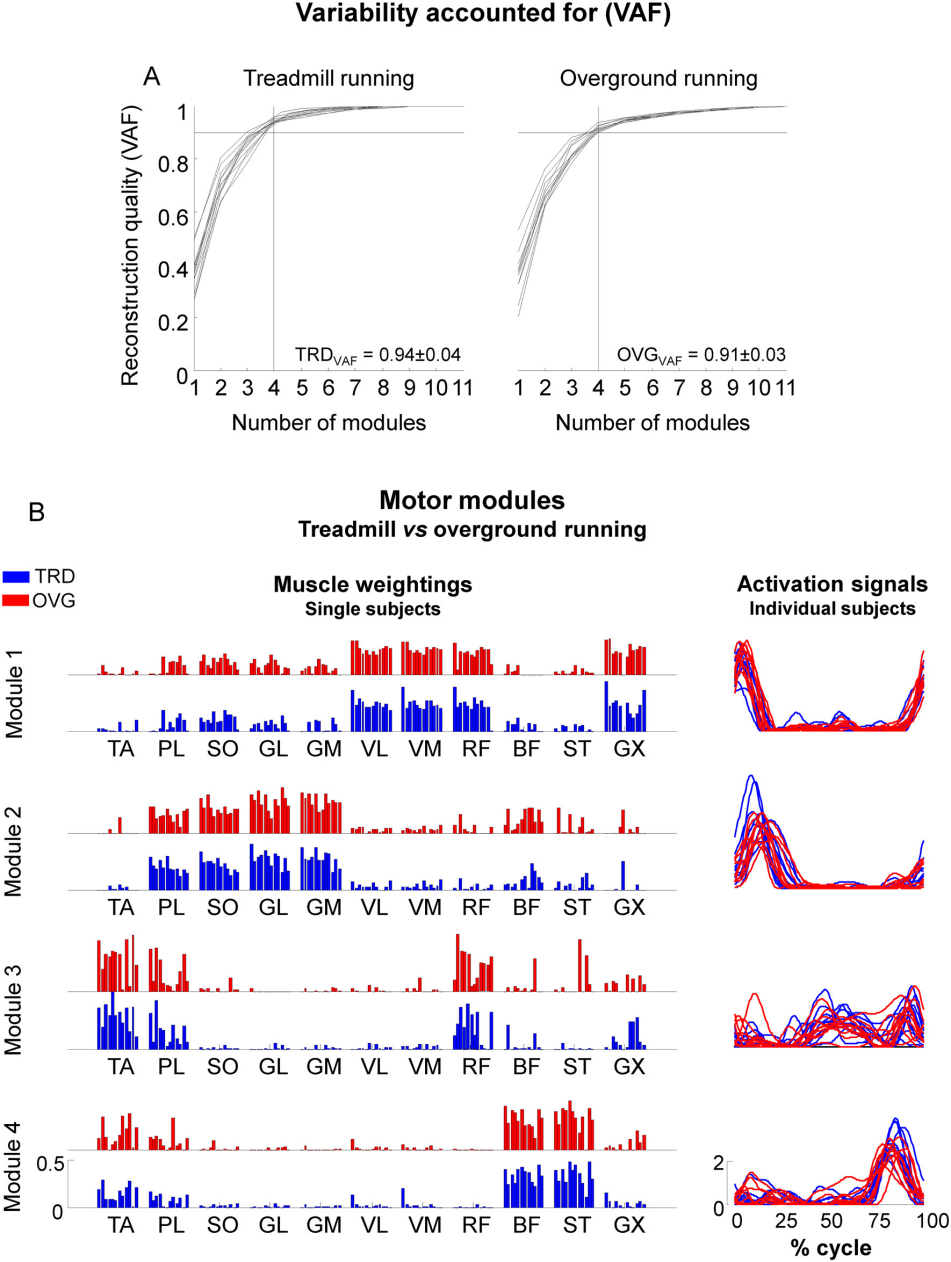
Motor modules—Running. Reconstruction quality of surface EMG signals (variation accounted for—VAF) by means of different number of motor modules (A) for each subject separately. The horizontal solid line in each panel corresponds to VAF at 0.9, and the vertical solid line correspond to the extraction of four motor modules. Mean±SD VAF for each condition and EMG processing method is displayed in the panels. We did not observe differences between locomotion conditions (treadmill *vs* overground running). In B, motor modules extracted from 20 consecutive gait cycles from treadmill running (*blue*) and overground running (*red*) are displayed for all subjects (*individual bars of the muscle weightings and lines of the activation signals*).

The analysis of dimensionality from concatenated trials revealed that four motor modules were required to reconstruct unilateral muscular activation for both treadmill and overground running with a reconstruction quality over 90% (Fig 4A, VAF = 92.5±3%).

### Motor modules representing treadmill and overground running

The four identified motor modules can be associated to fundamental biomechanical subtasks. From the representative subject reported in Fig 1, we observed that Module 1 (M1) consists of the activation of knee extensors and GX (see Fig 1B for illustration) at the transition from swing to stance (see Fig 1C for illustration). Module 2 (M2) relates to running propulsion, in which the plantar flexors are the predominant actuators. Module 3 (M3) relates to limb recovery, in which subjects recruited TA, PL and RF throughout the swing phase, and Module 4 (M4) is related to the activation of hamstrings (ST, BF) prior to landing. We recognized these specific subtasks and timing properties for most subjects for both TRD (*blue*) and OVG running (*red*, Fig 4B). However, visual inspection also indicated higher variability for the muscle weightings and activation signals in M3.

### Motor module similarities

**Fig 5:**
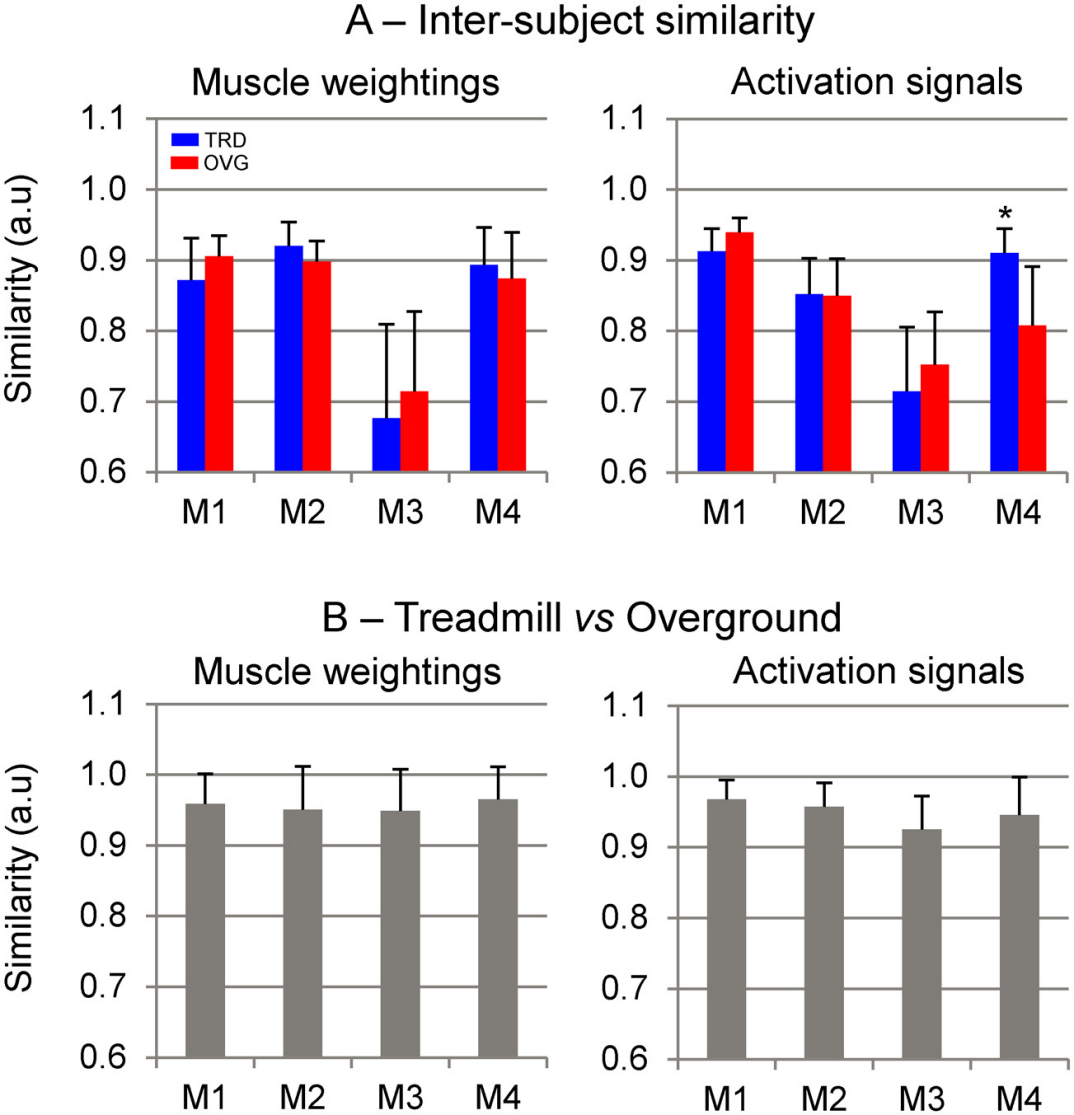
Motor modules—similarities. A: inter-subject similarities (Mean±SD) across the four identified muscle weightings and activation signals during treadmill running (*grey*) and overground running (*black*). B: intra-subject similarities between treadmill and overground running. * denotes significant difference in relation to overground running.

The inter-subject analysis revealed high similarities for muscle weightings of M1, M2 and M4 (0.87±0.1, Fig 5A), however, similarity was reduced for M3 (average similarity = 0.68±0.2, ~20% reduction). In the same way, there was a high similarity for activation signals of M1 and M2 (0.88±0.1), with reduced similarity for M3 (average similarity = 0.72±0.2, ~18% reduction).

We found no statistical differences between similarities from treadmill and overground running, except for the inter-subject similarity of the activation signals related to M4, for which we found a higher similarity for treadmill running (p<0.001). Similarities between treadmill and overground running were consistently high (>0.9 in average) for all muscle weightings and activation signals (Fig 5B).

### Curve subtractions and timing of motor modules

**Fig 6:**
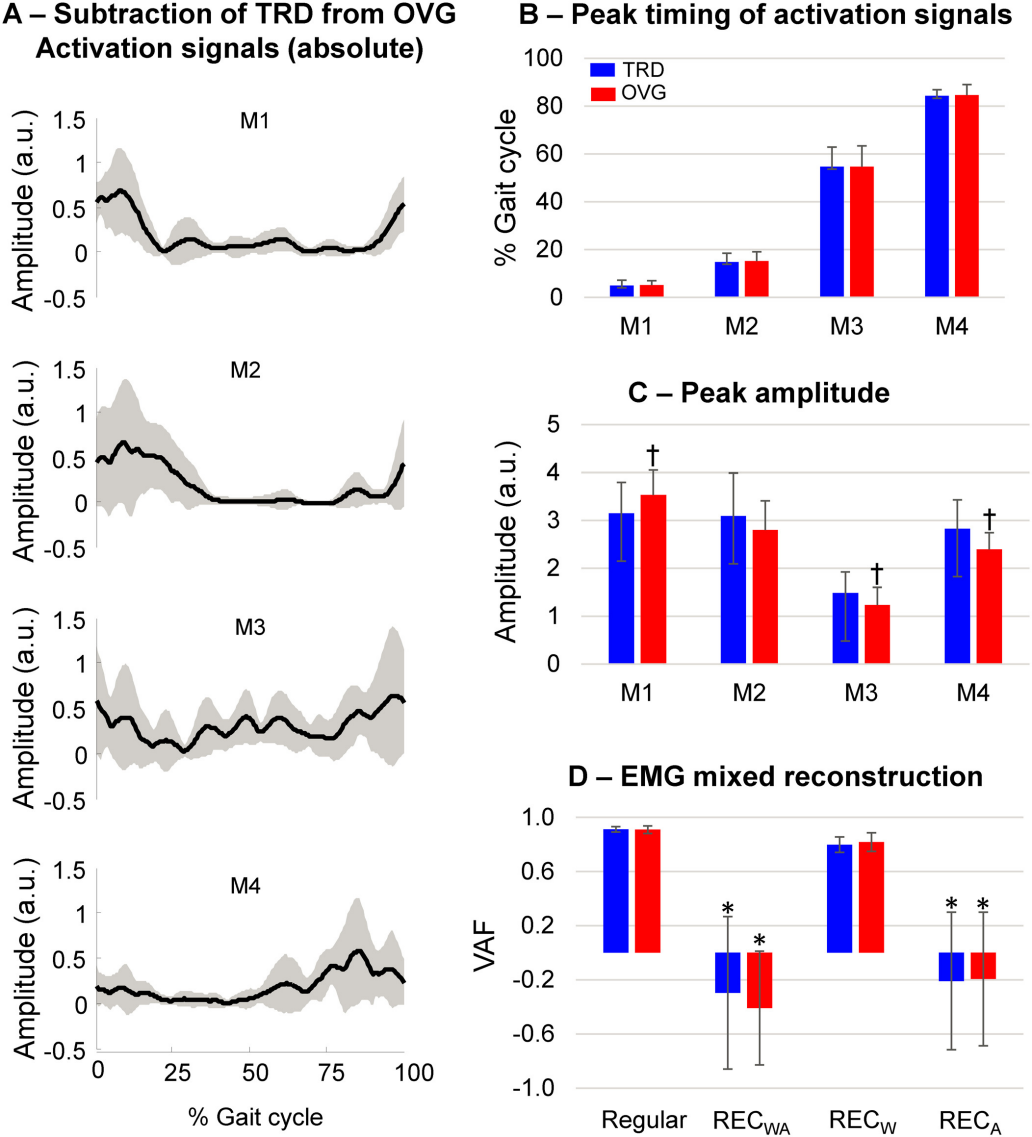
Modular organization—treadmill vs overground. A: Mean (*black line*) and SD (*gray area*) absolute subtraction of treadmill (TRD) from overground (OVG) activation signals. B: mean (SD) timing of the peak amplitude of activation signals from TRD and OVG running for each motor module (from M1 to M4). C: mean (SD) peak amplitude of activation signals for each motor module (from M1 to M4). D: reconstruction quality of multi-muscle EMG datasets from different combinations of muscle weightings and activation signals (please refer to Methods for explanation). † denotes significant difference in relation to TRD running (p<0.05). * denotes significant difference in relation to Regular and REC_W_ only (p<0.001).

Activation signals from M1 and M2 showed the greatest deviation between treadmill and overground running at the beginning of the gait cycle (Fig 6A). Following the results that M3 demonstrated the lowest similarities, the differences between activation signals from treadmill and overground running were spread throughout the entire gait cycle. For M4, the largest differences between conditions occurred at the end of the gait cycle. With respect to peak timing of activation signals, we found no significant differences between treadmill and overground running for all four motor modules (Fig 6B, p>0.05). On the other hand, there were significant differences in the magnitude of the peaks from activation signals (Fig 6C). We observed a significantly greater peak in M1 for overground in comparison to treadmill running (p<0.01), whereas peak amplitudes were lower in M3 and M4 for overground running (p<0.01).

### Reconstruction from mixed non-negative matrix factorization models

The reconstruction of EMG datasets showed, as expected, the highest quality by using muscle weightings and activation signals from their respective condition (Fig 6D). In addition, it was possible to successfully reconstruct about 80% of the EMG datasets by fixing the activation signals and using muscle weightings from any independent dataset (REC_W_). The reconstruction of EMG datasets by using both muscle weightings and activation signals from an independent dataset in REC_WA_ resulted in unsuccessful reconstruction. In the same way, by fixing muscle weightings and using activation signals from an independent dataset (REC_A_) the low VAF suggested that reconstruction was unsuccessful. Both these unsuccessful reconstructions were significantly lower than the regular reconstruction and the reconstruction performed by fixing activation signals and using muscle weightings from the other running dataset (p<0.001, F = 82.4).

## Discussion

The main findings of this study were that both treadmill and overground running share the same motor modules to perform biomechanical subtasks (load absorption, propulsion etc.). Both running conditions exhibit consistent peak timing for the burst-like activation of motor modules. However, the magnitude of the peaks related to initial contact indicated higher demands for running overground in comparison to running on the treadmill. At the same time, treadmill running induced greater magnitudes of peaks for leg swing and preparation to landing. Our results suggest that muscle activation during running under different environmental constraints is predominantly similar. Moreover, adjustments in muscle activation for each environment were based on the magnitude of the peak module activation rather than the timing.

### Changes in tibial acceleration

We found a higher variability for tibial acceleration during stance and preparation to landing for overground running in comparison to treadmill running. Movement stability during treadmill running might be compromised by changes in speed perception [40] and a reduction of optical flow [41]. Consequently, runners adopt a flatter foot position for landing, assuming a more cautious running style for optimizing stability [4,8,42]. On the other hand, overground running is performed over a stable surface and we speculate that runners may be more comfortable to vary lower limb muscle activation at initial contact. These suggestions can explain the higher variability for tibial acceleration during stance and preparation to landing during overground running. Previous investigations have shown reductions in peak vertical forces during treadmill running in comparison to overground running [2,5], while peak acceleration was not different between conditions in the present investigation. These contradictory results might be related to different instrumentation or protocols, as these previous studies used force sensors either on the floor or as insoles, whereas we recorded vertical tibial acceleration. In addition, differences in the experimental protocols such as the number of continuous running cycles, especially overground, could influence the outcomes impact forces. Riley et al. [2], performed overground tests on a 15-m runway and reported data from only three running strides. Garcia-Peres and co-workers [5] recorded overground data on a 400-m track but the data were equivalent to 3 seconds of recordings, which may provide 4–6 running cycles. Our results from 20 consecutive cycles suggest distinct tibial acceleration patterns during preparation to landing and stance period of treadmill and overground running with similar peak accelerations. More research using substantially higher number of continuous running cycles is needed for investigating overground running biomechanics.

### Individual EMG analysis

Previous studies investigating EMG differences between treadmill and overground running have focused on analyzing muscles separately. In this study we replicated such methodology using 11 lower limb muscles and also extracted motor modules from the same EMG datasets, in order to further explore the motor control perspective. We found reduced EMG activity during treadmill running for the ankle joint muscles (TA, PER and SO) using single-muscle analysis. These results corroborate a study from Baur and co-workers [8] who found reduced SO EMG during treadmill running, concomitant to longer PER EMG. The authors associated changes in SO EMG to mechanical requirements for running on a moving belt, which reduced the excitability of Golgi tendon organs from the muscle and consequently increases the afferent feedback. We also found reductions in TA peak EMG prior to landing, which may be necessary for controlling foot position towards safer initial contact as described previously [8,42].

### Motor modules—dimensionality and similarity

As expected [21], we found a similar number of modules representing the control of muscle activation during the two investigated environmental conditions. In addition to similar dimensionality, we found high inter-subject similarities between treadmill and overground running especially for M1 and M2 (stance phase). One previous study has also shown similar motor modules across different walking conditions [43], and several investigations have described similar motor modules across healthy individuals [35,44,45]. Human locomotion is modulated by a small set of basic patterned commands directed to the leg muscles [16], and some of these modules may be inborn from the evolution of vertebrates [14]. Therefore, the subjects in our experiment recruited similar synergies because running involves the recruitment of modules in a natural motor behavior with encoded motor patterns [7,37,46].

We found a reduced similarity between activation signals especially for the module related to the swing phase (M3). This reduction in similarity can be related to the changes in the overall shape of the activation curve for this module (Fig 6A). Consequently, these differences in M3 contributed to the failure in reconstructing EMG datasets from one condition (i.e., overground) using activation signals from another condition (i.e., treadmill). We speculate that these differences may be related to adjustments in muscle activation in order to cope with stride-to-stride differences at constant speed on the treadmill. A limitation may be that we only recorded a limited number of muscles related to hip flexion since surface EMG is limited for the recording of proximal hip muscles. This fact limits possible conclusions regarding the participation of deeper muscles not explored on this investigation on running.

### Peak timing and magnitude of activation signals

Our results showed that instances of peak activation of motor modules were similar between treadmill and overground running. On the other hand, the magnitude of these peaks varied. The time-invariant peak activation strongly supports the hypothesis of burst-like activation for the modulation of locomotor tasks [7]. Changes in the magnitude of peak activation between treadmill and overground running were the most prominent adaptations in the control of body displacement between the two running conditions. Increased magnitudes overground in M1 and the reduced magnitude M3 can be related to higher demands for load absorption in comparison to treadmill running. On the other hand, the reduced peak magnitudes for treadmill running in M3 may be related to the specific requirements for performing initial leg swing after pushing-off from a moving surface. We speculate that central commands from sensorimotor areas may regulate the magnitude of motor module activations to meet the requirements of the running surface, whereas muscle weightings are maintained. There are important contributions from supraspinal commands during locomotor tasks [23,47,48], and more research is needed to further elucidate the potential role of supraspinal inputs to the modulation of running under different environmental constraints.

### Limitations of the study

In the present study, we examined recreational runners performing at their preferred speed only. However, further investigations testing across different running speeds and also comparing recreational runners to elite runners are needed in order to advance our understanding on modular control of running. We provided careful instructions to subjects to not change running speed during recordings overground, however it is possible that the subjects performed small accelerations and decelerations throughout the 60 m corridor, but maintained a similar average speed. This might increase the inter-subject variability in EMG recordings for some muscles and prevented statistical comparisons to reach significance for the integrated EMG of muscles showing medium effect size (RF, BF and ST). This study was limited to the measurement of the muscles of the lower limb accessible by surface EMG, and the possible differences in muscle activation in the excluded muscles remains unclear. In order to understand the contribution of deeper muscles it would be required to use intramuscular EMG, while musculoskeletal modeling could also contribute to quantify muscle activation for a greater number of muscles. Finally, despite that the lack of kinematic measurements may be a limitation, there are several studies describing comparisons between treadmill and overground running [2,6,42], therefore we based our arguments on previous literature.

In summary, this study showed that treadmill and overground running share similar motor modules and timing for the predominant muscular peak activation. A lower variability in tibia acceleration and peak/integrated EMG during treadmill running suggests specific inertial requirements for maintaining smooth and effective running pattern and matching of the speed of the treadmill belt. Overground running required increased peak multi-muscle activity during preparation to landing, whereas peak magnitude of activation signals was higher for leg swing and preparation to landing during treadmill running. Differences in muscle activation can be described by temporal adjustments of specific motor modules, potentially assuring appropriate limb positioning and load absorption throughout several strides. The results from this study suggest that the varying environmental requirement of treadmill vs. overground running are fully attainable by a modular organisation of motor control.
